# All cause and cause specific mortality in obsessive-compulsive disorder: nationwide matched cohort and sibling cohort study

**DOI:** 10.1136/bmj-2023-077564

**Published:** 2024-01-17

**Authors:** Lorena Fernández de la Cruz, Kayoko Isomura, Paul Lichtenstein, Henrik Larsson, Ralf Kuja-Halkola, Zheng Chang, Brian M D’Onofrio, Isabell Brikell, Christian Rück, Anna Sidorchuk, David Mataix-Cols

**Affiliations:** 1 Centre for Psychiatry Research, Department of Clinical Neuroscience, Karolinska Institutet, SE-11330 Stockholm, Sweden; 2 Stockholm Health Care Services, Region Stockholm, Stockholm, Sweden; 3 Department of Medical Epidemiology and Biostatistics, Karolinska Institutet, Stockholm, Sweden; 4 School of Medical Sciences, Örebro University, Örebro, Sweden; 5 Department of Psychological and Brain Sciences, Indiana University, Bloomington, IN, USA; 6 Department of Global Public Health and Primary Care, University of Bergen, Bergen, Norway; 7 Department of Biomedicine, Aarhus University, Aarhus, Denmark; 8 Department of Clinical Sciences, Lund University, Lund, Sweden

## Abstract

**Objective:**

To estimate the risk of all cause and cause specific mortality in people with obsessive-compulsive disorder (OCD) compared with matched unaffected people from the general population and with their unaffected siblings.

**Design:**

Population based matched cohort and sibling cohort study.

**Setting:**

Register linkage in Sweden.

**Participants:**

Population based cohort including 61 378 people with OCD and 613 780 unaffected people matched (1:10) on sex, birth year, and county of residence; sibling cohort consisting of 34 085 people with OCD and 47 874 unaffected full siblings. Cohorts were followed up for a median time of 8.1 years during the period from 1 January 1973 to 31 December 2020.

**Main outcome measures:**

All cause and cause specific mortality.

**Results:**

4787 people with OCD and 30 619 unaffected people died during the study period (crude mortality rate 8.1 and 5.1 per 1000 person years, respectively). In stratified Cox proportional hazards models adjusted for birth year, sex, county, migrant status (born in Sweden versus abroad), and sociodemographic variables (latest recorded education, civil status, and family income), people with OCD had an increased risk of all cause mortality (hazard ratio 1.82, 95% confidence interval 1.76 to 1.89) and mortality due to natural causes (1.31, 1.27 to 1.37) and unnatural causes (3.30, 3.05 to 3.57). Among the natural causes of death, those due to endocrine, nutritional, and metabolic diseases, mental and behavioural disorders, and diseases of the nervous, circulatory, respiratory, digestive, and genitourinary systems were higher in the OCD cohort. Conversely, the risk of death due to neoplasms was lower in the OCD cohort compared with the unaffected cohort. Among the unnatural causes, suicide showed the highest hazard ratio, followed by accidents. The results were robust to adjustment for psychiatric comorbidities and familial confounding.

**Conclusions:**

Non-communicable diseases and external causes of death, including suicides and accidents, were major contributors to the risk of mortality in people with OCD. Better surveillance, prevention, and early intervention strategies should be implemented to reduce the risk of fatal outcomes in people with OCD.

## Introduction

Obsessive-compulsive disorder (OCD) is a usually chronic and disabling psychiatric disorder affecting about 2% of the population.^1^ ^2^ OCD is characterised by intrusive thoughts, urges, or images that trigger high levels of anxiety and other distressing feelings—known as obsessions—that the person tries to neutralise by engaging in repetitive behaviours or rituals—known as compulsions. These symptoms are time consuming and impairing and result in a poor quality of life.^3^ OCD is also associated with substantial socioeconomic adversity, including academic underachievement and labour market marginalisation.^4^ ^5^ In severe cases, people with OCD may struggle to maintain their personal hygiene, follow a healthy lifestyle, or even leave their home.^6^ ^7^ OCD has also been associated with substantial risk of alcohol and substance use disorders.^8^ All these factors can, in turn, increase the risk of a range of somatic diseases that have been associated with OCD.^9^

Data on the risk of mortality in OCD are scarce. A study carried out in the United States using data from the Epidemiologic Catchment Area Program reported that people with OCD (n=389, of whom 113 had died) had a 22% lower risk of death compared with unaffected people (adjusted proportional hazard ratio 0.78, 95% confidence interval 0.63 to 0.95).^10^ By contrast, a population based study in Denmark with a follow-up of nearly 10 years reported that the risk of mortality was approximately twofold higher in people with OCD (n=10 155, of whom 110 had died), compared with general population controls (mortality rate ratio 2.00, 95% confidence interval 1.65 to 2.40) and with their unaffected full siblings (1.87, 1.07 to 3.27).^11^ Unfortunately, these studies included too few outcomes to inspect specific causes of death. Some other studies have specifically focused on deaths due to suicide,^12^ ^13^ transport accidents,^14^ and substance use^8^; however, to the best of our knowledge, no studies have focused on specific natural causes of death in OCD. A better understanding of the specific causes of death in OCD, both natural and unnatural, would be clinically useful, as it would help to generate hypotheses about potential strategies for prevention and early intervention.

This population based cohort study linked prospectively collected data from the Swedish national registers over a 48 year period to describe the association between OCD and all cause and cause specific mortality in more than 60 000 people with a diagnosis of OCD, compared with matched individuals without OCD. We adjusted for a range of measured confounders, including sociodemographic variables and psychiatric comorbidities, given the associations between these variables and both the exposure and the outcomes under study.^1^ ^4^ ^5^ ^15^ ^16^ Additionally, to further adjust for unmeasured familial confounding, we used a sibling control design comparing people with OCD with their unaffected full siblings.

## Methods

### Study design and data sources

Using the unique national identification numbers assigned to Swedish citizens,^17^ we linked several Swedish population based registers. The National Patient Register includes diagnostic information on people admitted to a Swedish hospital since 1969, with complete data coverage for psychiatric disorders from 1973. From 2001, the register also contains data on outpatient consultations in specialised care.^18^ Diagnoses are based on ICD-8, ICD-9, and ICD-10 (international classification of diseases, eighth (1969-86), ninth (1987-96), and 10th revisions (1997 onwards). The Cause of Death Register contains a record of all deaths in Sweden since 1952, with compulsory reporting nationwide.^19^ Each record contains the date of death and codes for underlying and contributory causes of death, also using ICD codes. The Cause of Death Register covers more than 99% of all deaths in Swedish residents, including those occurring abroad, resulting in minimal loss of information. Sociodemographic data came from the Census Register, containing data from 1960, the Swedish Total Population Register, containing data on all Swedish inhabitants since 1968,^20^ and the Longitudinal Integration Database for Health Insurance and Labour Studies Register, which annually integrates data on the labour market, education, and social sectors from all people living in Sweden since 1990.^21^ Dates of immigration into and emigration out of Sweden came from the Migration Register.^20^ We identified full siblings via the Multi-Generation Register, which connects every person born in Sweden from 1932, and registered as living in the country from 1961, to their parents.^22^

### Matched cohort

We used a matched cohort design to estimate the risk of all cause and cause specific death in people with a diagnosis of OCD, compared with unaffected people from the general population. The study population included people who lived in Sweden at any time between 1973 and 2020 at the age of 6 years and older. We excluded people who died or emigrated before 1973 or their 6th birthday, whichever occurred last, and those who were born after 31 December 2014 (that is, less than six years before the study end on 31 December 2020).

We defined people with OCD as those who were given a diagnosis of OCD between 1 January 1973 and 31 December 2020 at the age of 6 years or older. They entered the cohort on the date of their first registered OCD diagnosis. We matched each person with OCD on sex, birth year, and county of residence at the time of the first OCD diagnosis with 10 unaffected people who had never had a diagnosis of OCD by the date of diagnosis of the person with OCD. To be eligible for matching, the unaffected people should be alive and living in Sweden at the age of 6 years or older at the date when their counterparts with OCD entered the cohort. We assigned them the same cohort entry date as the matched people with OCD. At the time of matching, we further excluded all people who had emigrated and then returned to Sweden between their 6th birthday or 1997, whichever came later, and the cohort entry date to allow for a more complete register coverage. We followed up people with OCD and the corresponding matched unaffected people from the cohort entry date until the date of the outcome (that is, death), emigration from Sweden, or the end of the study (that is, 31 December 2020), whichever occurred first. If unaffected people received a diagnosis of OCD after they had entered the cohort, they were also censored.

### Sibling cohort

We considered full siblings (that is, those sharing the same mother and father) of people with OCD for inclusion if they were singleton births, were living in Sweden between 1973 and 2020 at the age of 6 years and older, and had never received a diagnosis of OCD during the study period. We created family identification numbers to allow linkage between siblings with shared parents, making each family a stratum with differentially affected siblings. For family strata in which more than one sibling had a diagnosis of OCD, we chose one random sibling with OCD and all the unaffected siblings. We followed each sibling from 1 January 1973, the date of the 6th birthday, or date of immigration to Sweden, whichever came last, until the date of death, emigration from Sweden, or the end of the study period, whichever occurred first.

### Diagnosis

We identified people with a diagnosis of OCD, according to the Swedish ICD (ICD-8 code: 300.3; ICD-9: 300D; ICD-10: F42), from the National Patient Register as those having at least one record of inpatient or outpatient care between 1 January 1973 and 31 December 2020, if recorded at the age of 6 years or older, to avoid diagnostic misclassification.^14^ ^23^ ICD codes for OCD have been previously validated by comparing registered diagnoses with information from clinical records,^24^ showing excellent inter-rater reliability (κ=0.98) and moderate to excellent validity (positive predictive value of 0.55 in ICD-8, 0.64 in ICD-9, and 0.91-0.96 in ICD-10).

### Outcomes

For those people who died between 1973 and 2020, we extracted both all cause mortality data and, separately, the specific underlying cause of death from the Cause of Death Register. Specific causes of death were reported by group following the ICD chapters, provided that the number of deaths among the people with OCD was greater than 10 (to minimise the risk of identification of individuals). We combined the groups with small numbers of deaths and the causes of death classified as “codes for special purposes” in the ICD under an “other causes of death” category. Additionally, we grouped the specific causes of death into natural causes (all causes except those classified under the “external causes of morbidity and mortality” group) and unnatural causes (those classified as “external causes of morbidity and mortality”). We further divided this last category into accidents, suicides, and other unnatural causes of death. See supplementary table A for the specific groups and ICD codes.

### Covariates

For each study participant, we extracted country of birth (Sweden or abroad) from the Swedish Total Population Register. Additionally, we retrieved several sociodemographic variables from the Census Register or the Longitudinal Integration Database for Health Insurance and Labour Studies Register by using register records corresponding to the end of the follow-up (that is, latest registered). These included highest achieved level of education, categorised into elementary education (≤9 years), secondary education (10-12 years), and higher education (>12 years); civil status, classified into single, married or cohabiting, and divorced or widowed; and family income level, classified into lowest 20%, middle, and top 20%.

Information on psychiatric disorders other than OCD came from the National Patient Register. We grouped lifetime comorbid disorders into autism spectrum disorders, attention-deficit/hyperactivity disorder, and Tourette syndrome and chronic tic disorder (hereafter neurodevelopmental disorders); schizophrenia and other psychotic disorders (hereafter psychotic disorders); bipolar disorders; major depressive disorder, persistent mood disorder, and unspecified mood disorder (hereafter depressive disorders); phobic, anxiety, reaction to severe stress, and adjustment disorders (hereafter anxiety disorders); eating disorders; and substance use disorders. See supplementary table B for specific ICD codes.

### Statistical analyses

We calculated mortality rates per 1000 person years for affected and matched unaffected people. Additionally, we calculated the survival curves by exposure status by using Kaplan-Meier survival estimates.

We used stratified Cox proportional hazards regression analyses to estimate hazard ratios with 95% confidence intervals for time to death in people with a diagnosis of OCD, compared with matched unaffected people without a diagnosis of OCD, taking time in years after the cohort entry date as the underlying time scale. We did the analysis first for all causes of mortality as an outcome and then separately for each specific cause. The initial model (model 1—minimally adjusted model) adjusted for the matching variables, including birth year, sex, and county of residence at the time of the first OCD diagnosis (and at the corresponding time for the unaffected people) as covariates. A second model (model 2) additionally controlled for migrant status (born in Sweden versus abroad) and sociodemographic variables (that is, latest recorded education, civil status, and income). We marked missing data on these covariates as unknown and then included them in the Cox models as nominal variables. Given that OCD is slightly more prevalent in women than in men,^25^ and because some causes of death have shown differential risks by sex,^26^ ^27^ ^28^ we were interested in describing the associations of interest in men and in women, separately. Therefore, were repeated models 1 and 2 stratifying by sex. To explore the contribution of different groups of psychiatric comorbidities on the association between OCD and mortality, we repeated model 2 with additional adjustment for the above mentioned groups of psychiatric comorbidities, one group at a time (model 3).

We did two sensitivity analyses. Because the validity of the ICD-8 and ICD-9 codes for OCD in the National Patient Register is somewhat lower than that for the ICD-10 codes,^24^ we first repeated model 2 in a sub-cohort after excluding people who had died or emigrated from Sweden before 1997 (when the ICD-10 was implemented) and people with a diagnosis of OCD made using ICD-8 and/or ICD-9 codes only, as well as their corresponding matched unaffected people. In the second sensitivity analysis, we did a complete record analysis of model 2, in which we excluded people with missing data on the selected covariates.

Finally, to account for potential familial confounding, we compared the people with OCD with their unaffected full siblings. Stratified Cox proportional hazards regression models were adjusted first for sex, birth year, age at the start of follow-up for each sibling, and sibling order (model 1) and then additionally for migrant status and sociodemographic variables (education, civil status, family income; model 2). 

We used SAS version 9.4 for data management and analyses. All tests used two tailed significance set at P<0.05.

### Patient and public involvement

The board of the Swedish OCD Association (Svenska OCD-förbundet) reviewed our study plans and endorsed the research by providing a statement in grant applications. No individual patients were involved in the design of the study, the interpretation, or the write-up of results because this is a retrospective analysis of already collected data.

## Results

### Matched cohort description

We initially identified 16 121 414 people living in Sweden between 1 January 1973 and 31 December 2020. Of these, 15 557 510 were available for matching after exclusion of 563 904 people who had emigrated or died before age 6, were born after 31 December 2014, or had missing or conflicting county information. In this resulting cohort, 63 851 people had a diagnosis of OCD. We further excluded 2473 people who had emigrated between their 6th birthday or 1997 and the cohort entry date, had received a diagnosis before age 6, had conflicting diagnostic information (for example, diagnosis of OCD made after death or emigration), or had missing county information in the year of diagnosis. We matched the remaining 61 378 people with OCD one to 10 with 613 780 unaffected people on sex, birth year, and county.

Table 1 shows demographic and clinical characteristics of the population based cohorts. The median follow-up time was 8.1 (interquartile range 3.9-13.3) years. The median age at first diagnosis of OCD was 26.7 (18.4-38.7) years. People with OCD were more likely than the matched unaffected cohort to be born in Sweden, be less educated, be single, and have a lower family income. People with OCD also had higher rates of other lifetime psychiatric disorders, compared with unaffected people (85.7% *v* 19.6%).

**Table 1:** Baseline characteristics and follow-up data of study participants. Values are numbers (percentages) of participants unless stated otherwise

Characteristic	Population based matched cohort*			Sibling cohort*	
	People with OCD (n=61 378)	Matched unaffected controls (n=613 780)		People with OCD (n=34 085)	Unaffected full siblings (n=47 874)
Female sex	35 493 (57.8)	354 930 (57.8)		19 582 (57.5)	23 590 (49.3)
Country of birth:					
Sweden	54 824 (89.3)	508 781 (82.9)		32 720 (96.0)	45 700 (95.5)
Abroad	6554 (10.7)	104 999 (17.1)		1365 (4.0)	2174 (4.5)
Highest achieved level of education:					
Elementary education	14 771 (24.1)	102 952 (16.8)		7369 (21.6)	7340 (15.3)
Secondary education	24 349 (39.7)	244 966 (39.9)		13 592 (39.9)	19 045 (39.8)
Higher education	18 548 (30.2)	228 991 (37.3)		11 206 (32.9)	18 544 (38.7)
Unknown	3710 (6.0)	36 871 (6.0)		1918 (5.6)	2945 (6.2)
Civil status:					
Single	45 330 (73.9)	374 024 (60.9)		26 210 (76.9)	30 553 (63.8)
Married or cohabiting	10 808 (17.6)	188 581 (30.7)		5661 (16.6)	13 467 (28.1)
Divorced or widowed	3137 (5.1)	31 139 (5.1)		1067 (3.1)	1733 (3.6)
Unknown	2103 (3.4)	20 036 (3.3)		1147 (3.4)	2121 (4.4)
Family income level:					
Lowest 20%	24 357 (39.7)	122 961 (20.0)		10 003 (29.3)	6896 (14.4)
Middle	28 297 (46.1)	352 707 (57.5)		17 823 (52.3)	28 689 (59.9)
Top 20%	6843 (11.2)	117 221 (19.1)		5123 (15.0)	10 183 (21.3)
Unknown	1881 (3.1)	20 891 (3.4)		1136 (3.3)	2106 (4.4)
Any lifetime psychiatric comorbidities	52 586 (85.7)	120 493 (19.6)		27 549 (80.8)	12 624 (26.4)
Neurodevelopmental disorders	20 202 (32.9)	33 287 (5.4)		11 436 (33.6)	4497 (9.4)
Psychotic disorders	6636 (10.8)	6621 (1.1)		3421 (10.0)	851 (1.8)
Bipolar disorders	6618 (10.8)	7648 (1.2)		3676 (10.8)	1045 (2.2)
Depressive disorders	31 007 (50.5)	47 222 (7.7)		16 977 (49.8)	5716 (11.9)
Anxiety disorders	39 811 (64.9)	67 422 (11.0)		15 541 (45.6)	5180 (10.8)
Eating disorders	6534 (10.7)	10 115 (1.7)		3894 (11.4)	1156 (2.4)
Substance use disorders	11 529 (18.8)	30 976 (5.1)		6130 (18.0)	3531 (7.4)
Median (IQR) follow-up time, years	8.1 (3.9-13.1)	8.1 (3.9-13.3)		8.0 (3.9-13.0)	8.2 (4.0-13.4)
No of deaths (mortality per 1000 person years)	4787 (8.1)	30 619 (5.1)		1503 (4.7)	1261 (2.7)
Median (IQR) age at death, years	69.4 (53.9-80.9)	78.00 (65.8-86.6)		58.7 (44.4-69.1)	64.9 (52.6-74.1)

IQR=interquartile range; OCD=obsessive-compulsive disorder.

* All differences between individuals with OCD and matched unaffected individuals/siblings are significant at P<0.001, except for sex in the population based matched cohort (P=1.0), as it was used as a matching variable.

### All cause and cause specific mortality analyses

**Fig 1:**
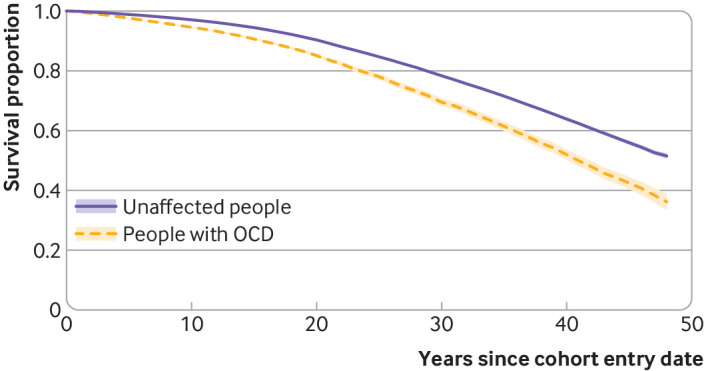
Kaplan-Meier survival curves (with 95% confidence intervals) in people with obsessive-compulsive disorder (OCD) and matched unaffected people

A total of 4787 people with OCD and 30 619 unaffected people died during the follow-up (crude mortality rate 8.1 and 5.1 per 1000 person years, respectively). Figure 1 shows the survival curves by OCD status during the follow-up period.

**Fig 2:**
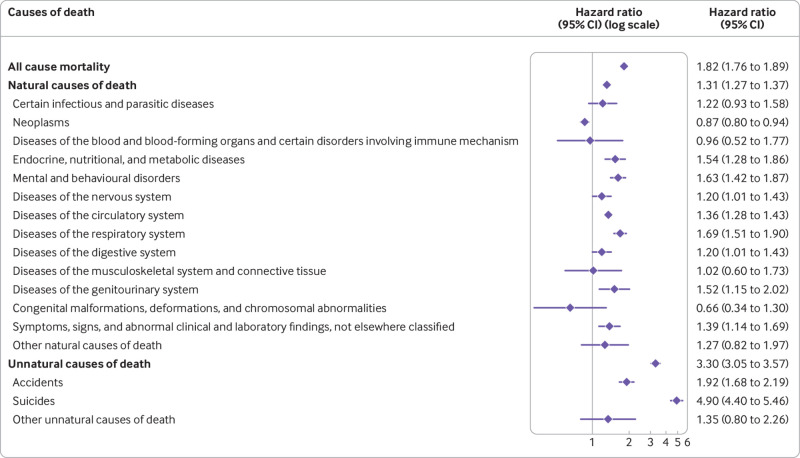
Hazard ratios adjusted for sex, birth year, county of residence, and migrant status and latest recorded education, civil status, and family income, with 95% confidence intervals (CIs), for all cause and cause specific mortality among people with obsessive-compulsive disorder compared with matched unaffected people

Minimally adjusted risk estimates (model 1) showed that people with OCD had a twofold risk of all cause mortality compared with the unaffected cohort (hazard ratio 2.00, 95% confidence interval 1.94 to 2.07) (table 2). This risk remained generally unchanged after adjustment for migrant status and all sociodemographic variables in model 2 (hazard ratio 1.82, 1.76 to 1.89) (fig 2). Model 2 estimates also showed an increased risk for both natural (hazard ratio 1.31, 1.27 to 1.37) and unnatural causes of death (3.30, 3.05 to 3.57). Furthermore, people with OCD, compared with unaffected people, had an increased risk of the following specific causes of death: endocrine, nutritional, and metabolic diseases; mental and behavioural disorders; diseases of the nervous system; diseases of the circulatory system; diseases of the respiratory system; diseases of the digestive system; diseases of the genitourinary system; symptoms, signs, and abnormal clinical laboratory findings; and external causes of morbidity and mortality. Among the last group, which represents the unnatural causes, the risks of death due to accidents and suicides were higher in people with OCD. Within the mental and behavioural disorders group, the most common specific causes of death were, in this order, unspecified dementia, vascular dementia, and mental and behavioural disorders due to use of alcohol, in both affected and unaffected people. The risk of death due to neoplasms was lower in people with OCD than in unaffected people (fig 2).

**Table 2:** Minimally adjusted hazard ratios with 95% confidence intervals (CIs) for all cause and cause specific mortality among people with obsessive-compulsive disorder (OCD), compared with matched unaffected people

Cause of death	No (%) people with OCD (n=61 381)	No (%) matched unaffected people (n=613 810)	Hazard ratio (95% CI)—model 1, minimally adjusted*
All cause mortality	4787 (7.80)	30 619 (4.99)	2.00 (1.94 to 2.07)
Natural causes of death	3707 (6.04)	28 302 (4.61)	1.53 (1.47 to 1.58)
Certain infectious and parasitic diseases	67 (0.11)	488 (0.08)	1.36 (1.06 to 1.76)
Neoplasms	781 (1.27)	8546 (1.39)	0.90 (0.83 to 0.97)
Diseases of the blood and blood-forming organs and certain disorders involving the immune mechanism	12 (0.02)	105 (0.02)	1.14 (0.63 to 2.07)
Endocrine, nutritional, and metabolic diseases	143 (0.23)	759 (0.12)	1.88 (1.57 to 2.24)
Mental and behavioural disorders	252 (0.41)	1475 (0.24)	1.72 (1.51 to 1.97)
Diseases of the nervous system	149 (0.24)	1135 (0.18)	1.31 (1.10 to 1.56)
Diseases of the circulatory system	1539 (2.51)	11 205 (1.83)	1.45 (1.38 to 1.53)
Diseases of the respiratory system	362 (0.59)	1935 (0.32)	1.88 (1.68 to 2.11)
Diseases of the digestive system	155 (0.25)	1057 (0.17)	1.45 (1.23 to 1.72)
Diseases of the musculoskeletal system and connective tissue	16 (0.03)	154 (0.03)	1.02 (0.61 to 1.72)
Diseases of the genitourinary system	59 (0.10)	352 (0.06)	1.66 (1.26 to 2.19)
Congenital malformations, deformations, and chromosomal abnormalities	18 (0.03)	105 (0.02)	1.68 (1.02 to 2.77)
Symptoms, signs, and abnormal clinical and laboratory findings, not elsewhere classified	124 (0.20)	763 (0.12)	1.64 (1.36 to 1.98)
Other natural causes of death†	30 (0.05)	223 (0.04)	1.33 (0.91 to 1.95)
Unnatural causes of death (external causes of morbidity and mortality)	1079 (1.76)	2303 (0.38)	4.77 (4.43 to 5.13)
Accidents	319 (0.52)	1184 (0.19)	2.66 (2.35 to 3.01)
Suicides	741 (1.21)	1007 (0.16)	7.44 (6.76 to 8.19)
Other unnatural causes of death	19 (0.03)	112 (0.02)	1.67 (1.02 to 2.71)

* Adjusted for all matching variables (sex, birth year, and county of residence at time of OCD diagnosis). Model 2 is reported in figure 2.

† Includes all groups with small number of deaths (≤10) in OCD cohort and causes of death classified in ICD as “codes for special purposes.”

Analyses stratified by sex and adjusted for birth year, county of residence, migrant status, and all sociodemographic variables (model 2) showed that women and men with OCD had a similar increase in risk of all cause mortality (hazard ratio 1.79 (1.71 to 1.88) and 1.83 (1.74 to 1.93), respectively) and of natural causes of death (1.31 (1.24 to 1.37) and (1.31 (1.23 to 1.38)), compared with their unaffected counterparts. By contrast, the risk of death due to unnatural causes was higher among women with OCD compared with unaffected women (hazard ratio 4.01, 3.55 to 4.53) than among men with OCD compared with unaffected men (2.87, 2.58 to 3.20) (supplementary tables C and D).

Further adjustment for psychiatric comorbidities (model 3) overall attenuated the magnitude of the risk of death estimates. For example, the estimates for the risk of all cause mortality now ranged from a hazard ratio of 1.53 (1.48 to 1.59) after adjustment for substance use disorders to 1.81 (1.74 to 1.87) after adjustment for eating disorders (table 3).

**Table 3:** Hazard ratios with 95% confidence intervals (CIs) for all cause and cause specific mortality among people with obsessive-compulsive disorder (OCD), compared with matched unaffected people, further adjusted for different groups of psychiatric comorbidities

Cause of death	Hazard ratio (95% CI) of model 3 plus additional adjustment for specific disorder group*						
	Neuro-developmental disorders	Psychotic disorders	Bipolar disorders	Depressive disorders	Anxiety disorders	Eating disorders	Substance use disorders
All cause mortality	1.74 (1.68 to 1.81)	1.64 (1.58 to 1.70)	1.72 (1.66 to 1.78)	1.55 (1.50 to 1.61)	1.61 (1.55 to 1.68)	1.81 (1.74 to 1.87)	1.53 (1.48 to 1.59)
Natural causes of death	1.29 (1.24 to 1.34)	1.22 (1.17 to 1.27)	1.27 (1.22 to 1.32)	1.19 (1.15 to 1.25)	1.23 (1.18 to 1.29)	1.31 (1.26 to 1.36)	1.17 (1.13 to 1.22)
Certain infectious and parasitic diseases	1.19 (0.91 to 1.55)	1.23 (0.94 to 1.62)	1.21 (0.93 to 1.59)	1.12 (0.84 to 1.49)	1.05 (0.78 to 1.40)	1.22 (0.94 to 1.59)	1.06 (0.81 to 1.39)
Neoplasms	0.86 (0.80 to 0.93)	0.85 (0.78 to 0.91)	0.86 (0.80 to 0.93)	0.86 (0.80 to 0.94)	0.83 (0.77 to 0.90)	0.87 (0.80 to 0.94)	0.83 (0.77 to 0.90)
Diseases of the blood and blood-forming organs and certain disorders involving the immune mechanism	0.94 (0.50 to 1.76)	0.94 (0.50 to 1.75)	0.87 (0.45 to 1.66)	0.83 (0.43 to 1.61)	1.01 (0.52 to 1.93)	0.96 (0.52 to 1.76)	0.97 (0.52 to 1.79)
Endocrine, nutritional, and metabolic diseases	1.48 (1.23 to 1.79)	1.48 (1.22 to 1.80)	1.44 (1.19 to 1.75)	1.30 (1.06 to 1.60)	1.38 (1.12 to 1.69)	1.54 (1.27 to 1.85)	1.39 (1.15 to 1.68)
Mental and behavioural disorders	1.61 (1.40 to 1.85)	1.43 (1.24 to 1.66)	1.53 (1.32 to 1.76)	1.45 (1.25 to 1.69)	1.44 (1.24 to 1.68)	1.56 (1.36 to 1.80)	1.39 (1.21 to 1.61)
Diseases of the nervous system	1.17 (0.98 to 1.40)	1.14 (0.95 to 1.37)	1.19 (1.00 to 1.43)	1.03 (0.85 to 1.24)	1.12 (0.93 to 1.36)	1.21 (1.01 to 1.44)	1.19 (0.99 to 1.42)
Diseases of the circulatory system	1.33 (1.26 to 1.41)	1.29 (1.22 to 1.37)	1.32 (1.25 to 1.40)	1.26 (1.19 to 1.34)	1.32 (1.24 to 1.40)	1.36 (1.28 to 1.44)	1.27 (1.20 to 1.34)
Diseases of the respiratory system	1.68 (1.49 to 1.89)	1.54 (1.36 to 1.73)	1.58 (1.40 to 1.78)	1.43 (1.26 to 1.62)	1.43 (1.26 to 1.62)	1.66 (1.48 to 1.87)	1.50 (1.33 to 1.69)
Diseases of the digestive system	1.18 (0.99 to 1.41)	1.15 (0.96 to 1.38)	1.18 (0.99 to 1.41)	1.03 (0.85 to 1.24)	1.05 (0.87 to 1.27)	1.19 (1.00 to 1.42)	0.85 (0.70 to 1.03)
Diseases of the musculoskeletal system and connective tissue	0.98 (0.57 to 1.69)	1.01 (0.59 to 1.74)	0.92 (0.53 to 1.60)	0.82 (0.46 to 1.44)	1.09 (0.62 to 1.91)	1.02 (0.60 to 1.73)	1.02 (0.60 to 1.74)
Diseases of the genitourinary system	1.52 (1.14 to 2.02)	1.34 (1.00 to 1.81)	1.37 (1.02 to 1.84)	1.24 (0.91 to 1.69)	1.33 (0.99 to 1.80)	1.52 (1.15 to 2.02)	1.50 (1.13 to 2.00)
Congenital malformations, deformations, and chromosomal abnormalities	0.69 (0.35 to 1.39)	0.62 (0.31 to 1.24)	0.72 (0.36 to 1.45)	0.74 (0.36 to 1.51)	0.70 (0.34 to 1.43)	0.66 (0.33 to 1.30)	0.68 (0.34 to 1.35)
Symptoms, signs, and abnormal clinical and laboratory findings, not elsewhere classified	1.30 (1.06 to 1.60)	1.30 (1.06 to 1.60)	1.35 (1.10 to 1.65)	1.18 (0.95 to 1.46)	1.12 (0.90 to 1.39)	1.40 (1.15 to 1.71)	1.21 (0.99 to 1.48)
Other natural causes of death†	1.16 (0.78 to 1.73)	1.27 (0.85 to 1.90)	1.12 (0.74 to 1.67)	0.83 (0.53 to 1.30)	0.92 (0.59 to 1.42)	1.20 (0.82 to 1.78)	1.18 (0.79 to 1.75)
Unnatural causes of death (external causes of morbidity and mortality)	2.98 (2.75 to 3.24)	2.89 (2.65 to 3.14)	2.96 (2.72 to 3.21)	2.17 (1.98 to 2.37)	2.19 (2.00 to 2.39)	3.19 (2.94 to 3.46)	2.53 (2.32 to 2.76)
Accidents	1.72 (1.50 to 1.98)	1.77 (1.54 to 2.04)	1.80 (1.57 to 2.26)	1.55 (1.34 to 1.79)	1.37 (1.18 to 1.59)	1.90 (1.66 to 2.17)	1.43 (1.24 to 1.65)
Suicides	4.43 (3.96 to 4.96)	4.14 (3.70 to 4.64)	4.27 (3.82 to 4.78)	2.68 (2.38 to 3.02)	2.95 (2.61 to 3.32)	4.66 (4.17 to 5.19)	3.75 (3.33 to 4.21)
Other unnatural causes of death	1.20 (0.70 to 2.08)	1.42 (0.82 to 2.45)	1.41 (0.83 to 2.39)	1.60 (0.91 to 2.84)	1.22 (0.70 to 2.14)	1.39 (0.82 to 2.35)	1.16 (0.67 to 1.99)

* Adjusted for all matching variables (sex, birth year, county of residence at time of OCD diagnosis), migrant status (Swedish born *v* born abroad), latest recorded highest level of education, family income level, and civil status, and specified group of psychiatric comorbidities.

† Includes all groups with small number of deaths (≤10) in OCD cohort and causes of death classified in ICD as “codes for special purposes.”

The sensitivity analysis excluding people with a diagnosis of OCD made using older ICD versions (ICD-8 and ICD-9) and the corresponding unaffected people showed similar results to those in the main analyses (hazard ratio for all cause mortality 1.83, 1.75 to 1.91; supplementary table E). Similarly, the results of the sensitivity analysis using complete records (that is, excluding people with missing data in the selected covariates) were similar to those in the main analysis (hazard ratio for all cause mortality 1.82, 1.76 to 1.89; supplementary tableF).

### Sibling analysis

Table 1 shows the characteristics of the sibling cohorts. The median follow-up time was 8.1 (interquartile range 3.9-13.2) years. Among all study participant with OCD, 34 085 people had 47 874 unaffected full siblings. People with OCD, compared with their unaffected full siblings, were more likely to be women, be born in Sweden, be less educated, be single, have a lower family income, and have a significantly higher rate of lifetime psychiatric comorbid disorders (table 1).

**Fig 3:**
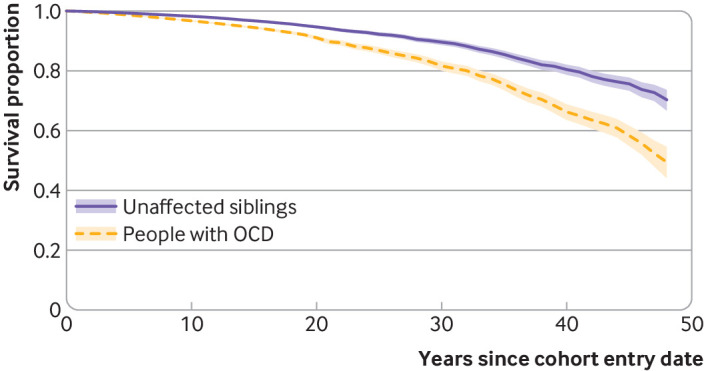
Kaplan-Meier survival curves (with 95% confidence intervals) in people with obsessive-compulsive disorder (OCD) and their unaffected siblings

A total of 1503 people with OCD and 1261 unaffected siblings had died during the follow-up, corresponding to a crude mortality rate of 4.7 and 2.7 per 1000 person years, respectively. Figure 3 shows the survival curves by OCD status during the follow-up period.

**Fig 4:**
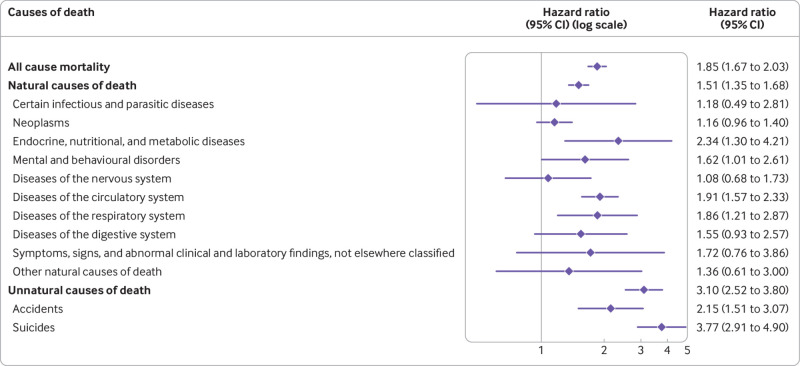
Hazard ratios adjusted for sex, birth year, age at start of follow-up for each sibling, sibling order, and migrant status and latest recorded education, civil status, and family income, with 95% confidence intervals (CIs), for all cause and cause specific mortality among people with obsessive-compulsive disorder compared with their unaffected full siblings

The adjusted risks (model 2) of all cause mortality (hazard ratio 1.85, 1.67 to 2.03), death from natural causes (1.51, 1.35 to 1.68), and death from unnatural causes (3.10, 2.52 to 3.80) were well in line with the risks observed in the main population based cohort analysis. Regarding the specific causes of death, the pattern also remained the same overall, but the estimates were less precise owing to limited power (table 4; fig 4).

**Table 4:** Hazard ratios with 95% confidence intervals (CIs) for all cause and cause specific mortality among people with obsessive-compulsive disorder (OCD), compared with unaffected full siblings

Cause of death	No (%) people with OCD (n=34 085)	No (%) unaffected full siblings (n=47 874)	Hazard ratio (95% CI)—model 1, minimally adjusted*
All cause mortality	1503 (4.41)	1261 (2.63)	2.26 (2.07 to 2.47)
Natural causes of death	1027 (3.01)	1054 (2.20)	1.80 (1.62 to 1.99)
Certain infectious and parasitic diseases	21 (0.06)	22 (0.05)	1.34 (0.64 to 2.81)
Neoplasms	274 (0.80)	415 (0.87)	1.19 (1.00 to 1.41)
Endocrine, nutritional, and metabolic diseases	57 (0.17)	40 (0.08)	2.70 (1.65 to 4.42)
Mental and behavioural disorders	66 (0.19)	46 (0.10)	2.21 (1.45 to 3.37)
Diseases of the nervous system	48 (0.14)	62 (0.13)	1.19 (0.78 to 1.83)
Diseases of the circulatory system	353 (1.04)	298 (0.62)	2.19 (1.83 to 2.63)
Diseases of the respiratory system	85 (0.25)	63 (0.13)	2.48 (1.72 to 3.59)
Diseases of the digestive system	59 (0.17)	49 (0.10)	2.02 (1.30 to 3.14)
Symptoms, signs, and abnormal clinical and laboratory findings, not elsewhere classified	29 (0.09)	24 (0.05)	1.78 (0.97 to 3.26)
Other causes of death†	35 (0.10)	35 (0.07)	1.36 (0.70 to 2.66)
Unnatural causes of death (external causes of morbidity and mortality)	476 (1.40)	207 (0.43)	4.09 (3.39 to 4.93)
Accidents	129 (0.38)	89 (0.19)	2.81 (2.04 to 3.86)
Suicides	345 (1.01)	112 (0.23)	5.05 (3.99 to 6.40)

* Adjusted for sex, birth year, age at start of follow-up for each sibling, and sibling order. Model 2 is reported in figure 4.

† Includes all groups with small number of deaths (≤10) in OCD or sibling cohorts and causes of death classified in the ICD as “codes for special purposes.”

## Discussion

In this population based matched cohort and sibling cohort study, people with OCD had a higher mortality rate than did matched unaffected people (8.1 *v* 5.1 per 1000 person years) and had an 82% increased risk of all cause mortality. The excess mortality was higher for both natural (31% increased risk) and, particularly, unnatural causes of death (3.3-fold increased risk). The risk of all cause mortality was similar in both women and men, although women with OCD had a higher relative risk of dying due to unnatural causes than did men with OCD, likely due to the lower baseline risk among women in the general population. Adjustment for different groups of psychiatric comorbidities attenuated the risks only slightly. The main results were also robust to adjustment for unmeasured familial confounding.

### Comparison with other studies

To the best of our knowledge, only two previous studies had specifically examined risk of all cause mortality in OCD and reached opposing conclusions.^10^ ^11^ Our all cause mortality estimates are in line with the results from Meier and colleagues in a Danish cohort study (n=10 155 cases of OCD) reporting that people with OCD had a twofold higher risk of dying than the general population.^11^ By contrast, Eaton and colleagues reported a protective effect of OCD for risk of death in a US based population survey.^10^ However, the survey was based on a small sample of cases and subjected to specific sampling methods. Results from nationwide register based studies are more likely to provide reliable and generalisable estimates.

We report, for the first time, on the specific causes of death in people with OCD, compared with unaffected people. Among the specific natural causes of death, people with OCD had increased risks due to respiratory system diseases (69% increased risk), mental and behavioural disorders (63%), endocrine, nutritional, and metabolic diseases (54%), diseases of the genitourinary system (52%), diseases of the circulatory system (36%), diseases of the nervous system (20%), and diseases of the digestive system (20%). Most of these causes can be classified as non-communicable diseases (for example, cardiovascular diseases, diabetes, chronic respiratory diseases, mental disorders, neurological disorders), which are often related to potentially modifiable behavioural risk factors. An unexpected finding in our study was that the risk of death due to neoplasms was 13% lower in the OCD cohort, compared with the matched unaffected people. A paradoxical observation has been previously described by which people with psychiatric conditions, including those with OCD,^29^ ^30^ ^31^ have lower or similar cancer incidence compared with unaffected people but higher cancer related mortality rates, likely attributed to lower detection rates in the psychiatric groups,^32^ ^33^ which we did not find in our study. These results are intriguing and should be replicated.

Among all causes of death, deaths due to unnatural causes had the highest risk estimates. This risk seemed to be mainly driven by a nearly fivefold increased risk of suicide. The increased risk of death by suicide in OCD has been previously reported at the population level by our group, using different methods and smaller albeit partially overlapping cohorts,^12^ ^13^ and others.^11^ Altogether, these more recent and methodologically sound results should settle previous claims that people with OCD have a relatively low risk of dying by suicide.^34^ ^35^ ^36^ The risk of death due to accidents was also higher in people with OCD than in unaffected people (92% increased risk). These findings extend previous work from our group showing a marginal risk of transport accidents in women with OCD,^14^ and they suggest that other kinds of accidents (for example, falls) may also be contributors to mortality in both women and men with OCD. More needs to be done to understand the reasons behind these excess deaths, as they are potentially preventable.

### Clinical and policy implications

Our results have clinical and public health implications. Non-communicable diseases and external causes of death, including suicides and accidents, were the most relevant contributors to the excess mortality in our OCD cohort. These causes of death can be conceptualised as preventable and/or treatable.^37^ Hence, prevention strategies, better surveillance, and early intervention strategies should be prioritised to avoid fatal outcomes. Specifically, prevention focused on the reduction of four major risk factors—namely, tobacco use, harmful use of alcohol, unhealthy diet, and physical inactivity—is thought to be crucial for the control of non-communicable diseases.^38^ Furthermore, modification of lifestyle factors in people at risk has also been suggested as an effective strategy to prevent cognitive impairment and dementia.^39^ Significant efforts should also be made to promote early detection and improve access to specialist treatment for people with OCD. People with psychiatric disorders are known to be less likely to seek help for health related problems and to attend medical check-ups, and they are also less likely to receive health interventions and prescriptions for non-psychiatric drugs, potentially leading to delays in the detection and treatment of diseases.^32^ ^33^ ^40^ ^41^ A better integration of mental and physical healthcare services, the prioritisation of prevention while strengthening treatment, and the optimisation of intervention synergies across social-ecological levels have been recently proposed as priority action points to prevent premature mortality in people with psychiatric disorders.^42^

### Strengths and limitations of study

The study had some notable strengths. To the best of our knowledge, this is the largest study of mortality in people with OCD to date. We followed up a cohort of more than 60 000 people with OCD, of whom almost 5000 had died, and corresponding matched unaffected people throughout the study period. Additionally, this is the first study to be sufficiently powered to systematically investigate specific natural causes of death in OCD. The ICD codes for OCD have excellent reliability and good validity.^24^ The Swedish Cause of Death Register has an outstanding nationwide coverage.^19^ We systematically adjusted for lifetime psychiatric comorbidities and ruled out potential familial confounding. 

However, some limitations should be considered when interpreting the results. Firstly, the National Patient Register includes only diagnoses made in specialist care, with information from outpatient care available only from 2001 onwards, which may have resulted in the inclusion of people with OCD at the more severe end of the spectrum. Secondly, the frequency of some of the specific causes of death was relatively low, particularly in the sibling cohort, which may have led to underpowered estimates for those specific outcomes. Thirdly, our second analytical model adjusted for, among other covariates, the last registered level of education, civil status, and family income level, and a third model additionally adjusted for psychiatric comorbidities, one at a time. These covariates could potentially act as mediators rather than confounders, most likely driving the association estimates down when controlled for. However, we took a parsimonious approach including also a minimally adjusted model that did not include sociodemographic variables or comorbidities, and the results were broadly comparable. Fourthly, variables known to be associated with several non-communicable diseases (for example, sedentary lifestyle, unhealthy diet, smoking) were not available in the registers, so we could not explore their effect on the associations. Finally, the study was based in Sweden, and whether our findings generalise to other settings with different populations, with different health systems and medical practices, and where discrepancies in lifestyle behaviours and use of services may exist is unclear.

### Conclusions

In this population based matched cohort and sibling cohort study, non-communicable diseases and external causes of death, including suicides and accidents, were major contributors to the risk of mortality in people with OCD. Better surveillance, prevention, and early intervention strategies should be implemented to reduce the risk of fatal outcomes in people with OCD.

**What is already known on this topic**

Obsessive-compulsive disorder (OCD) has been associated with an increased risk of mortality
Previous studies on specific causes of death in OCD have mainly focused on unnatural causes (eg, suicide), but little is known about specific natural causes

**What this study adds**

People with OCD had an increased risk of death due to a wide range of natural causes of death, many of which can be classed as non-communicable and preventable
People with OCD had a lower risk of death due to neoplasms
External causes of death, including suicides and accidents, are major contributors to the risk of death in OCD

## Data Availability

No additional data available. The Public Access to Information and Secrecy Act in Sweden prohibits individual level data being publicly available. Researchers who are interested in replicating this study can apply for individual level data through Statistics Sweden (https://www.scb.se/en/services/ordering-data-and-statistics/ordering-microdata/) and the National Board of Health and Welfare (https://www.socialstyrelsen.se/en/statistics-and-data/registers/).

## Web extra

Extra material supplied by authors

Web appendix: Supplementary tables

## Notes

Contributors: LFC and DMC conceived the study. LFC, KI, AS, and DMC designed the methods. LFC wrote the first draft of the manuscript. KI did the analyses and produced the original figures. PL, HL, RKH, ZC, BMD, and IB contributed to the methods. All authors contributed to the interpretation of the results and critical revision of the manuscript for important intellectual content and approved the final version. LFC and KI are the guarantors. The corresponding author attests that all listed authors meet authorship criteria and that no others meeting the criteria have been omitted.

The lead author (the manuscript’s guarantor) affirms that this manuscript is an honest, accurate, and transparent account of the study being reported; that no important aspects of the study have been omitted; and that any discrepancies from the study as planned (and, if relevant, registered) have been explained.

Dissemination to participants and related patient and public communities: As this is a register based study based on de-identified data, the results of the study will not be fed back to individual patients but will be widely disseminated among members of the Swedish OCD Association and other international interest organisations as well as social media. The research findings will be widely disseminated to the wider community through press releases, social media platforms, presentations at international forums, and reports to relevant government agencies, academic societies, and national and international interest organisations.

Provenance and peer review: Not commissioned; externally peer reviewed.
