# Supplementary material for: All cause and cause specific mortality in obsessive-compulsive disorder: nationwide matched cohort and sibling cohort study
Source: BMJ. 2024 Jan 17;384:e077564. doi: 10.1136/bmj-2023-077564 (PMC10792686; doi:10.1136/bmj-2023-077564)
Supplement: Supplementary file 1 — Web appendix: Supplementary tables [file ferl077564.ww1.pdf]

SUPPLEMENTARY MATERIAL

Supplementary Table A. Swedish International Classification of Diseases (ICD) codes used to group specific causes of death in the study

Specific cause of death	Corresponding Swedish ICD-8 diagnostic codes (deaths from 1973 to 1986)	Corresponding Swedish ICD-9 diagnostic codes (deaths from 1987 to 1996)	Corresponding Swedish ICD-10 diagnostic codes (deaths from 1997 to 2020)
**Certain infectious and parasitic diseases**	000-136	001-139	A00−B99
**Neoplasms**	140-239	140-239	C00−D48
**Diseases of the blood and blood-forming organs and certain disorders involving the immune mechanism**	275, 280-289	279, 280-289	D50−D89
**Endocrine, nutritional, and metabolic diseases**	240-279, minus 275	240-279, minus 279	E00−E90
**Mental and behavioral disorders**	290-315	290-319	F00−F99
**Diseases of the nervous system**	320-324, 330-333, 340-349, 350-358	320-326, 330-337, 340-349, 350-359	G00−G99
**Diseases of the eye and adnexa**[a]	360-369, 370-379	360-379	H00−H59
**Diseases of the ear and mastoid process**[a]	380-389	380-389	H60−H95
**Diseases of the circulatory system**	390-458	390-459	I00−I99
**Diseases of the respiratory system**	460-519	460-519	J00−J99
**Diseases of the digestive system**	520-577	520-579	K00−K93
**Diseases of the skin and subcutaneous tissue**[a]	680-709	680-709	L00−L99
**Diseases of the musculoskeletal system and connective tissue**	710-738	710-739	M00−M99
**Diseases of the genitourinary system**	580-629	580-629	N00−N99
**Pregnancy, childbirth, and the**	630-678	630-679	O00−O99

**puerperium**[a]			
**Certain conditions originating in the perinatal period**	760-779	760-779	P00−P96
**Congenital malformations, deformations and chromosomal abnormalities**	740-759	740-759	Q00−Q99
**Symptoms, signs and abnormal clinical and laboratory findings, not elsewhere classified**	780-796	780-799	R00−R99
**Codes for special purposes**	–	–	U00-U99
**External causes of morbidity and mortality**	E807-E999<br><br>**Accidents:**<br>E807-E949<br>**Suicides:**<br>E950-E959, E980-E989	E800-E999<br><br>**Accidents:**<br>E800-E929<br>**Suicides**:<br>E950-E959, E980-E989	V01−Y98<br><br>**Accidents:**<br>V01-X59<br>**Suicides**:<br>X60-X84, Y10-34

[a]Grouped together under 'Other causes of death' given the small number of deaths due to this cause in the study.

Supplementary Table B. Swedish International Classification of Diseases (ICD) codes used to identify lifestime diagnoses of psychiatric disorder comorbidities in the study

Psychiatric disorders	Corresponding Swedish ICD-8 diagnostic codes	Corresponding Swedish ICD-9 diagnostic codes	Corresponding Swedish ICD-10 diagnostic codes
**Neurodevelopmental disorders:** Autism spectrum disorders, attention-deficit/ hyperactivity disorder[a], and Tourette syndrome and chronic tic disorder[b]	306.2	299, 307C, 314	F84, F90, F95
**Psychotic disorders:** Schizophrenia and other psychotic disorders	295 (minus 295.5), 297, 298 (minus 298.09)	295 (minus 295F), 297, 298 (minus 298A)	F20, F21, F22, F23, F24, F25 (minus F25.0), F28, F29
**Bipolar disorders**	296 (minus 296.00 and 296.20)	296 (minus 296B)	F25.0, F30, F31, F34.0
**Depressive disorders:** Major depressive disorder, persistent mood disorder, and unspecified mood disorder	296.0, 296.2, 298.09	296B, 298A, 300E, 311	F32, F33, F34 (minus F34.0), F38, F39
**Anxiety disorders:** Phobic, anxiety, reaction to severe stress, and adjustment disorders	300.0, 300.2, 307, 308.4	300A, 300C, 308, 309	F40.0, F40.1, F40.2, F41.0, F41.1, F43
**Eating disorders**	–	307B, 307F	F50.0-F50.3, F50.9
**Substance use disorder**	303, 304	303, 304, 305A, 305X	F10-F16, F18-19

[a]Individuals with attention-deficit/hyperactivity disorder (ADHD) were also identified by prescription of ADHD drugs, collected from the Prescription Drug Register, specifically Amphetamine (Anatomical Therapeutic Chemical [ATC] Classification System code: N06BA01), Dexamphetamine (N06BA02), Methylphenidate (N06BA04), Atomoxetine (N06BA09), and Lisdexamphetamine (N06BA12). [b]Tourette syndrome and chronic tic disorder were identified following the algorithm described in Rück et al.[24]

Supplementary Table C. Hazard ratios (HRs) with 95% confidence intervals (CIs) for all cause and cause specific mortality among women with obsessive-compulsive disorder (OCD), compared with matched unaffected women

Causes of death	Women with OCD (N=35,493) n (%)	Matched unexposed women (N=354,930) n (%)	HR (95% CI) Model 1[a]	HR (95% CI) Model 2[b]
**All-cause mortality**	2,544 (7.17)	17,341 (4.89)	1.88 (1.80 to 1.97)	1.79 (1.71 to 1.88)
**Natural causes of death**	2,073 (5.84)	16,469 (4.64)	1.48 (1.41 to 1.55)	1.31 (1.24 to 1.37)
Certain infectious and parasitic diseases	34 (0.10)	268 (0.08)	1.27 (0.89 to 1.82)	1.24 (0.87 to 1.79)
Neoplasms	462 (1.30)	4,835 (1.36)	0.94 (0.85 to 1.03)	0.90 (0.82 to 1.00)
Endocrine, nutritional and metabolic diseases	74 (0.21)	407 (0.11)	1.83 (1.43 to 2.35)	1.51 (1.17 to 1.96)
Mental and behavioural disorders	158 (0.45)	1,031 (0.29)	1.56 (1.32 to 1.84)	1.54 (1.30 to 1.83)
Diseases of the nervous system	77 (0.22)	714 (0.20)	1.09 (0.86 to 1.38)	1.01 (0.80 to 1.29)
Diseases of the circulatory system	809 (2.28)	6,496 (1.83)	1.31 (1.22 to 1.41)	1.26 (1.17 to 1.36)
Diseases of the respiratory system	233 (0.66)	1,114 (0.31)	2.12 (1.84 to 2.44)	1.94 (1.68 to 2.25)
Diseases of the digestive system	75 (0.21)	571 (0.16)	1.30 (1.03 to 1.66)	1.12 (0.88 to 1.44)
Diseases of the genitourinary system	36 (0.10)	209 (0.06)	1.72 (1.21 to 2.45)	1.61 (1.12 to 2.31)
Symptoms, signs and abnormal clinical and laboratory findings, not elsewhere classified	67 (0.19)	456 (0.13)	1.48 (1.15 to 1.92)	1.37 (1.05 to 1.78)
Other natural causes of death[c]	48 (0.14)	368 (0.10)	1.29 (0.95 to 1.74)	1.14 (0.84 to 1.56)
**Unnatural causes of death**				
External causes of morbidity and mortality	471 (1.33)	865 (0.24)	5.46 (4.88 to 6.11)	4.01 (3.55 to 4.53)
Accidents	121 (0.34)	476 (0.13)	2.50 (2.05 to 3.06)	2.10 (1.71 to 2.58)
Suicides	342 (0.96)	345 (0.10)	9.82 (8.45 to 11.4)	6.39 (5.39 to 7.58)

[a]Adjusted for all matching variables (i.e., sex, birth year, county of residence at the time of OCD diagnosis). [b]Adjusted for all variables in Model 1 and additionally for migrant status (Swedish born *v* born abroad) and latest recorded highest level of education, family income level, and civil status. [c]Includes all groups with a small number of deaths (≤10) in the OCD cohort for at least one of the sexes and the causes of death classified in the ICD as 'codes for special purposes'.

Supplementary Table D. Hazard ratios (HRs) with 95% confidence intervals (CIs) for all cause and cause specific mortality among men with obsessive-compulsive disorder (OCD), compared with matched unexposed men

Causes of death	Men with OCD (N=25,885) n (%)	Matched unexposed men (N=258,550) n (%)	HR (95% CI) Model 1[a]	HR (95% CI) Model 2[b]
**All-cause mortality**	2,243 (8.67)	13,278 (5.13)	2.15 (2.05 to 2.26)	1.83 (1.74 to 1.93)
**Natural causes of death**	1,634 (6.31)	11,833 (4.57)	1.59 (1.51 to 1.68)	1.31 (1.23 to 1.38)
Certain infectious and parasitic diseases	33 (0.13)	220 (0.08)	1.47 (1.02 to 2.13)	1.15 (0.79 to 1.69)
Neoplasms	319 (1.23)	3,711 (1.43)	0.84 (0.75 to 0.95)	0.82 (0.73 to 0.92)
Endocrine, nutritional and metabolic diseases	69 (0.27)	352 (0.14)	1.93 (1.49 to 2.49)	1.53 (1.16 to 2.00)
Mental and behavioural disorders	94 (0.36)	444 (0.17)	2.09 (1.68 to 2.62)	1.77 (1.41 to 2.24)
Diseases of the nervous system	72 (0.28)	421 (0.16)	1.68 (1.31 to 2.16)	1.52 (1.17 to 1.97)
Diseases of the circulatory system	730 (2.82)	4,709 (1.82)	1.65 (1.52 to 1.79)	1.46 (1.34 to 1.58)
Diseases of the respiratory system	129 (0.50)	821 (0.32)	1.57 (1.30 to 1.89)	1.36 (1.13 to 1.65)
Diseases of the digestive system	80 (0.31)	486 (0.19)	1.63 (1.28 to 2.06)	1.27 (0.99 to 1.62)
Diseases of the genitourinary system	23 (0.09)	143 (0.06)	1.58 (1.02 to 2.45)	1.41 (0.90 to 2.23)
Symptoms, signs and abnormal clinical and laboratory findings, not elsewhere classified	57 (0.22)	307 (0.12)	1.88 (1.41 to 2.49)	1.35 (1.00 to 1.83)
Other natural causes of death[c]	28 (0.11)	219 (0.08)	1.26 (0.85 to 1.87)	0.91 (0.60 to 1.38)
**Unnatural causes of death**				
External causes of morbidity and mortality	608 (2.35)	1438 (0.56)	4.34 (3.94 to 4.78)	2.87 (2.58 to 3.20)
Accidents	198 (0.76)	708 (0.27)	2.77 (2.36 to 3.24)	1.81 (1.52 to 2.15)
Suicides	399 (1.54)	662 (0.26)	6.17 (5.44 to 6.99)	4.09 (3.55 to 4.72)

[a]Adjusted for all matching variables (i.e., sex, birth year, county of residence at the time of OCD diagnosis). [b]Adjusted for all variables in Model 1 and additionally for migrant status (Swedish born *v* born abroad) and latest recorded highest level of education, family income level, and civil status. [c]Includes all groups with a small number of deaths (≤10) in the OCD cohort for at least one of the sexes and the causes of death classified in the ICD as 'codes for special purposes'.

Supplementary Table E. Hazard ratios (HRs) with 95% confidence intervals (CIs) for all cause and cause specific mortality among people with obsessive-compulsive disorder (OCD) diagnosed using ICD-10 codes, compared with matched unaffected people

Causes of death	Individuals with OCD (N=57,703) n (%)	Matched unexposed individuals (N=576,799) n (%)	HR (95% CI) Model 1[a]	HR (95% CI) Model 2[b]
**All-cause mortality**	2,699 (4.68)	14,546 (2.52)	2.12 (2.03 to 2.22)	1.83 (1.75 to 1.91)
**Natural causes of death**	1,947 (3.37)	13,054 (2.26)	1.64 (1.56 to 1.73)	1.47 (1.40 to 1.55)
Certain infectious and parasitic diseases	41 (0.07)	265 (0.05)	1.54 (1.10 to 2.14)	1.29 (0.92 to 1.82)
Neoplasms	423 (0.73)	4,376 (0.76)	0.95 (0.86 to 1.05)	0.92 (0.83 to 1.02)
Endocrine, nutritional and metabolic diseases	83 (0.14)	405 (0.07)	2.03 (1.60 to 2.57)	1.64 (1.28 to 2.10)
Mental and behavioural disorders	138 (0.24)	685 (0.12)	2.02 (1.68 to 2.43)	1.79 (1.49 to 2.16)
Diseases of the nervous system	102 (0.18)	650 (0.11)	1.56 (1.26 to 1.92)	1.38 (1.11 to 1.71)
Diseases of the circulatory system	706 (1.22)	4,383 (0.76)	1.63 (1.50 to 1.77)	1.50 (1.38 to 1.63)
Diseases of the respiratory system	184 (0.32)	872 (0.15)	2.09 (1.78 to 2.45)	1.76 (1.49 to 2.07)
Diseases of the digestive system	103 (0.18)	532 (0.09)	1.93 (1.56 to 2.39)	1.49 (1.20 to 1.86)
Diseases of the genitourinary system	33 (0.06)	139 (0.02)	2.32 (1.59 to 3.40)	2.11 (1.42 to 3.13)
Congenital malformations, deformations, and chromosomal abnormalities	15 (0.03)	72 (0.01)	2.06 (1.18 to 3.59)	0.78 (0.34 to 1.78)
Symptoms, signs and abnormal clinical and laboratory findings, not elsewhere classified	84 (0.15)	416 (0.07)	2.01 (1.59 to 2.55)	1.58 (1.23 to 2.02)
Other natural causes of death[c]	21 (0.04)	142 (0.02)	1.33 (0.93 to 1.89)	1.22 (0.85 to 1.75)
**Unnatural causes of death**				
External causes of morbidity and mortality	752 (1.30)	1,492 (0.26)	5.03 (4.61 to 5.49)	3.27 (2.97 to 3.61)
Accidents	234 (0.41)	700 (0.12)	3.28 (2.83 to 3.81)	2.17 (1.85 to 2.56)
Suicides	509 (0.88)	719 (0.12)	7.07 (6.31 to 7.92)	4.75 (4.18 to 5.38)

[a]Adjusted for all matching variables (i.e., sex, birth year, county of residence at the time of OCD diagnosis). [b]Adjusted for all variables in Model 1 and additionally for migrant status (Swedish born *v* born abroad) and latest recorded highest level of education, family income level, and civil status. [c]Includes all groups with a small number of deaths (≤10) in the OCD cohort and the causes of death classified in the ICD as 'codes for special purposes'.

Supplementary Table F. Hazard ratios (HRs) with 95% confidence intervals (CIs) for all cause and cause specific mortality among people with obsessive-compulsive disorder (OCD), compared with matched unaffected people, excluding people with missing data in the selected covariates

	**Individuals with OCD (N=57,659)**	**Matched unexposed individuals (N=575,645)**	**HR (95% CI) Model 1[a]**	**HR (95% CI) Model 2[b]**
**Causes of death**	**n (%)**	**n (%)**		
**All-cause mortality**	4,287 (7.44)	27,622 (4.80)	2.02 (1.95 to 2.09)	1.82 (1.76 to 1.89)
**Natural causes of death**	3,365 (5.84)	25,529 (4.43)	1.62 (1.56 to 1.69)	1.50 (1.44 to 1.56)
Certain infectious and parasitic diseases	65 (0.11)	472 (0.08)	1.42 (1.10 to 1.85)	1.24 (0.95 to 1.63)
Neoplasms	708 (1.23)	7,718 (1.34)	0.93 (0.86 to 1.01)	0.95 (0.87 to 1.02)
Diseases of the blood and blood-forming organs and certain disorders	11 (0.02)	96 (0.02)	1.18 (0.63 to 2.21)	0.98 (0.51 to 1.86)
Endocrine, nutritional and metabolic diseases	133 (0.23)	699 (0.12)	1.97 (1.64 to 2.38)	1.62 (1.33 to 1.96)
Mental and behavioural disorders	235 (0.41)	1,429 (0.25)	1.74 (1.51 to 2.00)	1.58 (1.37 to 1.82)
Diseases of the nervous system	140 (0.24)	1,091 (0.19)	1.31 (1.09 to 1.56)	1.19 (0.99 to 1.43)
Diseases of the circulatory system	1,359 (2.36)	9,817 (1.71)	1.54 (1.45 to 1.64)	1.44 (1.35 to 1.53)
Diseases of the respiratory system	330 (0.57)	1,747 (0.30)	1.96 (1.74 to 2.21)	1.72 (1.52 to 1.94)
Diseases of the digestive system	147 (0.25)	964 (0.17)	1.60 (1.35 to 1.91)	1.31 (1.10 to 1.58)
Diseases of the musculoskeletal system and connective tissue	16 (0.03)	137 (0.02)	1.19 (0.71 to 2.00)	1.16 (0.68 to 1.99)
Diseases of the genitourinary system	56 (0.10)	322 (0.06)	1.77 (1.33 to 2.35)	1.60 (1.19 to 2.15)
Congenital malformations, deformations, and chromosomal abnormalities	16 (0.03)	90 (0.02)	1.79 (1.05 to 3.05)	0.65 (0.31 to 1.34)
Symptoms, signs and abnormal clinical and laboratory findings, not elsewhere classified	119 (0.21)	730 (0.13)	1.70 (1.40 to 2.07)	1.40 (1.15 to 1.72)
Other natural causes of death[c]	30 (0.05)	217 (0.04)	1.39 (0.95 to 2.04)	1.25 (0.84 to 1.84)
**Unnatural causes of death**				
External causes of morbidity and mortality	921 (1.60)	2,079 (0.36)	4.53 (4.19 to 4.90)	3.21 (2.95 to 3.49)
Accidents	295 (0.51)	1,075 (0.19)	2.77 (2.43 to 3.16)	2.00 (1.74 to 2.30)
Suicides	607 (1.05)	897 (0.16)	6.88 (6.20 to 7.63)	4.77 (4.26 to 5.34)
Other unnatural causes of death	19 (0.03)	107 (0.02)	1.74 (1.06 to 2.83)	1.39 (0.83 to 2.35)

[a]Adjusted for all matching variables (i.e., sex, birth year, county of residence at the time of OCD diagnosis). [b]Adjusted for all variables in Model 1 and additionally for migrant status (Swedish born *v* born abroad) and latest recorded highest level of education, family income level, and civil status. [c]Includes all groups with a small number of deaths (≤10) in the OCD cohort and the causes of death classified in the ICD as 'codes for special purposes'.
